# Relationship between cardiac diffusion tensor imaging parameters and anthropometrics in healthy volunteers

**DOI:** 10.1186/s12968-015-0215-0

**Published:** 2016-01-06

**Authors:** L.A. McGill, P.F. Ferreira, A.D. Scott, S. Nielles-Vallespin, A. Giannakidis, P.J. Kilner, P.D. Gatehouse, R. de Silva, D.N. Firmin, D.J. Pennell

**Affiliations:** 1 NIHR Cardiovascular Biomedical Research Unit, Royal Brompton Hospital, Sydney Street, London, SW3 6NP UK; 2 National Heart and Lung Institute, Imperial College, London, UK; 3 National Heart, Lung and Blood Institute, National Institutes of Health, Bethesda, MD USA

**Keywords:** Diffusion tensor imaging, Fractional anisotropy, Mean diffusivity, Eigenvector, Helix angle, Helix angle gradient, Sheetlet

## Abstract

**Background:**

In vivo cardiac diffusion tensor imaging (cDTI) is uniquely capable of interrogating laminar myocardial dynamics non-invasively. A comprehensive dataset of quantative parameters and comparison with subject anthropometrics is required.

**Methods:**

cDTI was performed at 3T with a diffusion weighted STEAM sequence. Data was acquired from the mid left ventricle in 43 subjects during the systolic and diastolic pauses. Global and regional values were determined for fractional anisotropy (FA), mean diffusivity (MD), helix angle gradient (HAg, degrees/%depth) and the secondary eigenvector angulation (E2A). Regression analysis was performed between global values and subject anthropometrics.

**Results:**

All cDTI parameters displayed regional heterogeneity. The RR interval had a significant, but clinically small effect on systolic values for FA, HAg and E2A. Male sex and increasing left ventricular end diastolic volume were associated with increased systolic HAg. Diastolic HAg and systolic E2A were both directly related to left ventricular mass and body surface area. There was an inverse relationship between E2A mobility and both age and ejection fraction.

**Conclusions:**

Future interpretations of quantitative cDTI data should take into account anthropometric variations observed with patient age, body surface area and left ventricular measurements. Further work determining the impact of technical factors such as strain and SNR is required.

## Background

The cardiac musculature has a unique structure with myocytes winding progressively from a left-handed helix in the epicardium, to circumferential in the mid wall, to a right handed-helix in the endocardium [1–3]. The link between this structure and cardiac function is complex, and as yet not fully understood. During systolic contraction wall thickness increases by 30–50% radially, however this cannot be fully accounted for by myocyte thickening of ~8% [4–6], or changes in helix angle alone [1, 7]. There is increasing evidence that laminar myocardial structures, called sheetlets, provide a further contribution to wall thickening through systolic reorientation from the longitudinal to the radial plane [6, 8–11]. Furthermore reduced sheetlet mobility has been linked with myocardial hypertrophy in hypertrophic cardiomyopathy [8]. However, there remains a paucity of in vivo data assessing the dynamic function of myocytes and sheetlets in healthy hearts.

Diffusion tensor imaging (DTI) is an MRI technique, which enables novel in vivo interrogation of biological tissue structures noninvasively. DTI exploits the fact that tissue components, such as cell walls and collagen, act as natural barriers to diffusion resulting in anisotropy in the measured diffusion. When diffusion sensitizing gradients are applied, the degree of signal attenuation is dependent on the orientation of the gradient, with the greatest attenuation observed when gradients are applied parallel to the long axis of cells i.e. the orientation of greatest diffusion [12]. By measuring diffusion in 6 or more non-collinear directions a diffusion ellipsoid, or diffusion tensor, can be calculated, which represents the anisotropic diffusion in 3 dimensions. A number of quantitative parameters describing the orientation and arrangement of tissue microstructures can be derived from this tensor [13].

DTI is an established technique in central nervous system imaging [14–17] but there is increasing interest in cardiac DTI (cDTI) and a number of studies have validated cDTI findings against myocardial histology [18–20]. The primary eigenvector (the principal diffusion direction) aligns with the long axis of myocytes, thus allowing the helical structure of the myocardium to be characterised [21]. Laminar structures, such as sheetlets and shear layers, tend to lie oblique to the local wall plane; therefore the direction of the secondary eigenvector of diffusion (E2A) is thought to align with the principal population of sheetlets in a voxel [10, 11, 22–25]. In light of its complex structure, historically the left ventricular (LV) myoarchitecture has proven remarkably resistant to functional assessment. In vivo cDTI is therefore uniquely capable of combining non-invasive interrogation of laminar structures with functional analysis.

In vivo cDTI remains challenging in light of the difficulty of measuring small scale diffusion in the face of myocardial strain and bulk cardiac motion, nonetheless our group has established that the in vivo cDTI STEAM sequence has both good inter and intra-centre reproducibility [26–28]. There is also a growing body of evidence that cardiovascular disease processes result in detectable differences in quantitative cDTI parameters [8, 29–33], however the relationship between healthy subject anthropometrics and cDTI parameters has yet to be established. In this work we examine the relation between laminar myocardial structures, via quantitative cDTI parameters, and anthropometrics within a cohort of healthy volunteers.

## Methods

### Study population

We prospectively recruited 46 healthy volunteers for cDTI. Subjects were screened and excluded in the event of the following: significant co-morbidities; risk factors for ischaemic heart disease; high blood pressure; or an abnormal ECG. The study was approved by the National Research Ethics Service Committee South East Coast-Surrey, and was conducted in accordance with the principles set out in the declaration of Helsinki, with written informed consent obtained from all volunteers.

### Image acquisition

Images were acquired using a clinical 3T scanner (Magnetom Skyra, Siemens AG Healthcare Sector, Erlangen, Germany) with an anterior 18 element matrix coil and 8–12 elements of a matrix spine coil. Functional and volumetric data was determined from breath hold, retrospectively gated bSSFP cine acquisition in three long axis planes and contiguous short axes from the atrioventricular ring to the apex [34]. The timing of subject specific systolic and diastolic pauses were determined from a high temporal resolution, retrogated cine in the mid- ventricular short axis plane. cDTI data were acquired in held expiration with a monopolar diffusion weighted stimulated echo sequence (DW-STEAM), triggered on every other R wave [26]. The acquisition time was shortened with parallel imaging and a reduced field of view in the phase encoding direction [35]. Data was acquired in 3 mid ventricular slices during the systolic and diastolic pauses. Slice position was tracked between systole and diastole with a breath-hold spoiled gradient echo (GRE) sequence with a spatial modulation of magnetization (SPAMM) tagging prepulse in the 2 and 4 chamber views. The linear tags were separated by 16 mm and were perpendicular to the long-axis with an acquired spatial resolution of 2.1 × 1.7 mm in-plane and slice thickness of 6 mm. The displacement of the linear tag closest to the central mid- ventricular slice was manually tracked from the systolic to the diastolic phase.

The following sequence parameters were used, as previously described [8, 26, 27]: reference plus 6 diffusion encoding directions, fat saturation, TR = 2 RR intervals = 2000 ms (assuming a heart rate of 60 beats per minute), TE = 23 ms, BW = 2442 Hz/pixel, GRAPPA parallel imaging acceleration factor of 2 [36], field of view = 360 × 135 mm^2^, slice thickness 8 mm, EPI echo train length = 24 readouts, EPI readout duration = 13 ms, in-plane spatial resolution = 2.8 × 2.8 mm^2^ interpolated to 1.4 × 1.4 mm^2^, by zero filling, 3 slices, 4 mm slice gap. For diffusion encoding, the maximum available on axis gradient strength of 45 mT/m was used with a trapezoidal gradient pulse duration of 10 ms, leading to a diffusion sensitivity of b = 350 s/mm^2^ (assuming a heart rate of 60 beats per minute). When acquiring the reference images, spoiler gradients are used in place of the diffusion encoding gradients. These gradients introduce a diffusion weighting (135 s/mm^2^ in this protocol) to the reference images which was accounted for when calculating the diffusion tensor. Localized first and second-order shimming and frequency adjustment were performed with an adjustment box positioned to cover the region of interest. To improve the signal to noise ratio, the acquisition was repeated a minimum of 8 times, with up to 13 repetitions when one or more acquisitions were affected by motion artefacts from breathing or ectopy. Breath-hold duration was 18 cardiac cycles, which was typically 18 s. The order that the diffusion directions were acquired was also rotated to avoid losing data from the same direction at the end of each breath hold. The typical duration of the scan was 45 min.

### Diffusion tensor analysis

All cDTI data was analysed by a single observer with in-house custom-built software written in MATLAB (Mathworks, MA, USA). This included a quality control step to reject images visually corrupted by artefacts and rigid co-registration of the remaining images [37]. The RR-intervals during the acquisition were calculated from the acquisition times recorded in the DICOM files. The b-value of each diffusion image was then adjusted with this information. The eigensystem (eigenvalues and eigenvectors) was calculated for each voxel from a rank 2 diffusion tensor using the “B-matrix approach” described by Kingsley [38]. Infrequently, eigenvalues were measured as negative, and when these were present, they were set to the mean of the corresponding non-negative eigenvalues in neighbouring voxels.

Two scalar diffusion parameter maps were calculated from the eigensystems: Fractional Anisotropy (FA) and Mean Diffusivity (MD) [13]. FA is an index of the degree of deviation of the observed diffusion from isotropic and ranges from 0 (completely isotropic) to 1 (completely anisotropic), and MD measures the average diffusivity of the tensor. The primary, or largest eigenvector (E1) of the diffusion tensor was taken to represent mean intravoxel myocycte orientation [18–20]. The helix angle (HA) was calculated by projecting E1 radially to the local wall tangent plane. HA was defined as the angle in this plane between the E1 projection and the circumferential direction in the range −90 to 90°, being positive (right-handed helix) if rotated counter-clockwise from the circumferential as viewed from the outside, and negative (left-handed helix) if rotated clockwise [22]. To obtain a single value relating to helix angle, a gradient was calculated from best-fit radial projections drawn from the centre of the ventricle to the epicardial border. The gradient data are presented in two formats: HAG (degrees/mm) and HAg (degrees/%depth). Both HAG [28] and HAg [9] have previously been employed in HA analyses, with the latter permitting analysis of true angular change with a reduced dependence on wall thickness.

The secondary eigenvector (E2A) was taken to represent the mean sheetlet orientation [22–25]. The cross-myocyte plane, perpendicular to E1, was calculated for each voxel. The secondary eigenvector angle (E2A) was taken as the angle between E2A and the cross-myocyte direction. This angle was measured in the range 0–90, with zero degrees as being aligned to the local LV wall. E2A mobility was defined as the change of mean absolute E2A values from diastole to systole. E2A mobility uses the absolute value of the angle so polarity is ignored to provide a measure of change of angulation.

Global values for FA, MD, HAg and E2A were calculated in systole and diastole. . The myocardium was then divided into 4 segments for each slice and the same segments in each slice were grouped into the conventional LV walls (anterior, septal, inferior, lateral) [39]. Care was taken to exclude papillary muscle regions and septal contributions from the right ventricle. The post-processing and analysis time was approximately 1 h per subject.

### Image analysis

Ventricular volumes, function, mass, and ejection fraction for all patients were measured for the LV using a semi-automated threshold-based technique (CMRtools, Cardiovascular Imaging Solutions, London). All volume and mass measurements were also indexed to body surface area [40, 41].

### Statistical analysis

Statistical analysis was performed using IBM SPSS Statistics software, version 22. Cohort characteristics are presented as the mean ± SD or median & range, and percentage for categorical data. Variations in parameters between slices were assessed, with no discernible between slice difference for FA, MD and HAg; global and regional data are thus presented from all three slices to reduce parameter variation. However, despite a small interspace gap, there were significant between slice variations in E2A data, therefore global and regional wall E2A data are presented from the mid slice only. Comparison of regional data was conducted with a one-way repeated measures ANOVA, with correction for non-spherical data. Post hoc analysis was performed with paired t-tests with Bonferroni correction, taking the lateral wall as the reference.

The following covariates were analysed for association with cDTI measurements: age, gender, body surface area (BSA), ejection fraction (EF), left ventricular end diastolic volume (LVEDV), left ventricular end systolic volume (LVESV), LV mass and maximum LV wall thickness in diastole (MWTd). The association between covariates and global systolic and diastolic quantitative cDTI parameters were assessed with simple linear regression with significance set at <0.05. For helix angle data regression was performed for both HAg and HAG to determine whether normalising the gradient to the local wall thickness eliminates anthropometric associations.

## Results

### Study population

The baseline anthropometrics and CMR data of the study population are shown in Table 1. A total of 46 volunteers were recruited, 2 were excluded due to ECG irregularities, and 1 further subject was excluded due to poor quality diastolic data. Therefore data are presented for 43 subjects.

**Table 1:** Baseline characteristics mean ± SD, or median & range

Baseline Characteristics	*N* = 43
Age: (years &range)	45 (24–74)
Male subjects	26 (60%)
RR interval (ms)	960 ± 160
BSA: (m^2^)	1.88 ± 0.20
BMI: (kg/m^2^)	24.5 ± 3.0
LVEDV: (mL)	146 ± 33
LVESV: (mL)	47 ± 13
LVM: (g)	128 ± 32
MWTd (mm)	9 ± 1.2
LVEF: (%)	68 ± 6

*BSA* body surface area, *BMI* body mass index, *LVEDV* Left ventricular end diastolic volume, *LVESV* Left ventricular end systolic volume, *LVM,* Left ventricular mass, *MWTd* Maximum wall thickness in diastole, *LVEF* left ventricular ejection fraction

### Global and regional cDTI results

#### Fractional anisotropy

**Fig. 1:**
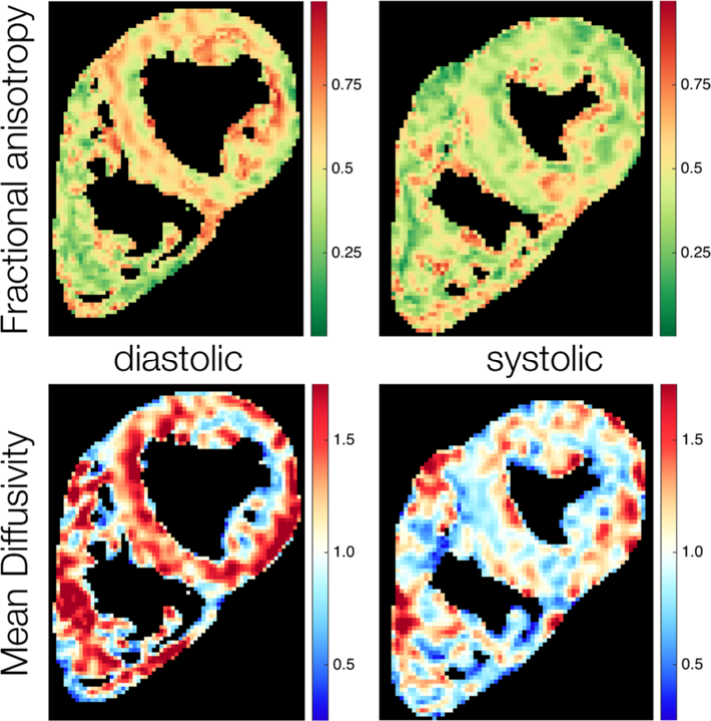
Example parameter maps of fractional anisotropy and mean diffusivity in systole and diastole. Minor regional heterogeneity is observed in both FA and MD

A comparison of regional systolic and diastolic FA values is given in Table 2. An example parameter map of FA in systole and diastole is given in Fig. 1. The global systolic FA was 0.47 ± 0.05. With the lateral wall as the reference, the FA was greater in the inferior (*p* = 0.007) and anterior (*p* = 0.017) walls. Global diastolic FA was 0.56 ± 0.04. The lateral wall FA was less than the anterior (*p* < 0.001), inferior (*p* < 0.001) and septal walls (*p* = 0.004).

**Table 2:** Regional fractional anisotropy in systole and diastole, mean ± SD

Fractional Anisotropy	*N* = 43	*p* value
Systolic:		ANOVA: F = 5.21, *p* = 0.003
Lateral	0.46 ± 0.05	Reference
Anterior	0.48 ± 0.05	*p* = 0.017
Inferior	0.48 ± 0.06	*p* = 0.007
Septal	0.46 ± 0.07	*p* = 1.0
Diastolic:		ANOVA: F = 16.0, *p* <0.001
Lateral	0.52 ± 0.07	Reference
Anterior	0.59 ± 0.05	*p* < 0.001
Inferior	0.59 ± 0.05	*p* < 0.001
Septal	0.54 ± 0.09	*p* = 0.004

Values are Bonferroni corrected and therefore a p value of *p* < 0.05 was taken as the level of significance

#### Mean diffusivity

Regional systolic and diastolic MD values are shown in Table 3, an example parameter map of MD in systole and diastole is shown in Fig. 1. Global systolic MD was 0.93 ± 0.14 × 10^3^ mm^2^/s. There were no regional differences in systolic MD (*p* = 0.22). Global diastolic MD was 1.11 ± 0.13 × 10^3^ mm^2^/s. The lateral wall MD was significantly greater than the anterior (*p* < 0.001) and inferior walls (*p* = 0.01).

**Table 3:** Regional mean diffusivity (×10^3^ mm^2^/s) in systole and diastole, mean ± SD

Mean Diffusivity (×10^3^ mm^2^/s)	*N* = 43	*p* value
Systolic:		ANOVA: F = 1.50, *p* = 0.22
Lateral	0.95 ± 0.16	
Anterior	0.90 ± 0.18	
Inferior	0.93 ± 0.18	
Septal	0.94 ± 0.18	
Diastolic:		ANOVA: F = 14.3, *p* <0.001
Lateral	1.20 ± 0.24	Reference
Anterior	0.99 ± 0.15	*p* <0.001
Inferior	1.05 ± 0.18	*p* = 0.01
Septal	1.20 ± 0.25	*p* = 1.0

Values are Bonferroni corrected and therefore a p value of *p* < 0.05 was taken as the level of significance

#### Helix angle gradient

**Fig. 2:**
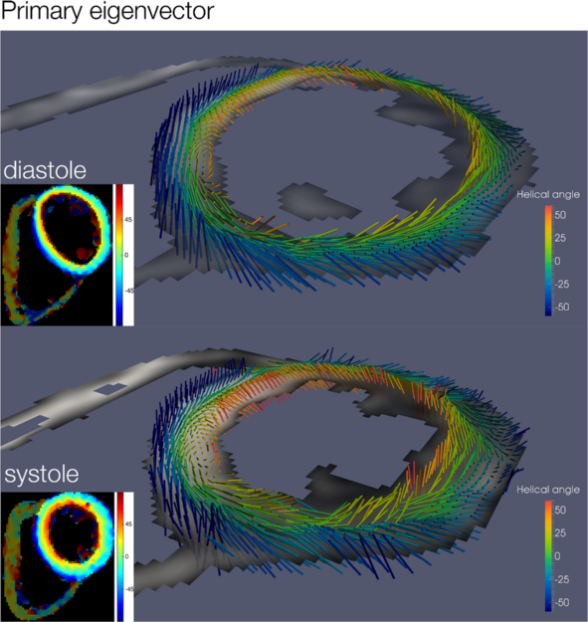
3D representation of HA (primary eigenvector) in diastole and systole showing a progression of myocytes from a left handed helix in the epicardium (*blue*), to circumferential in the mesocardium (*green*) to a right handed helix in the endocardium (*red*)

**Fig. 3:**
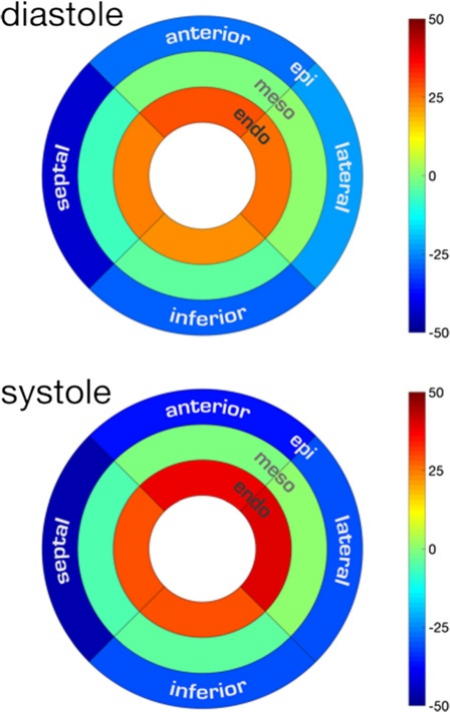
Bullseye plot of the average helix angle per LV regional wall and transmural layer (mean ± SD) in both systole and diastole

The helix angle in systole and diastole is depicted in 3D in Fig. 2. Transmural and regional helix angle values are represented in a Bullseye plot in Fig. 3. Regional, systolic and diastolic HAg values are displayed in Table 4. The global systolic HAg was 0.91 ± 0.11°. In comparison to the lateral wall HAg was greater in the septum (*p* = 0.007) and less in the inferior wall (*p* < 0.001). Global diastolic HAg was 0.68 ± 0.1°. HAg was greater in the anterior wall (*p* < 0.001) and the septum (*p* < 0.001) compared to the lateral wall.

**Table 4:** Regional Helix angle gradient (HAg, °/%) in systole and diastole, mean ± SD

Helix Angle Gradient(°/%)	*N* = 43	*p* value
Systolic:		ANOVA: F = 24.0, *p* < 0.001
Lateral	0.90 ± 0.16	Reference
Anterior	0.98 ± 0.21	*p* = 0.13
Inferior	0.76 ± 0.15	*p* < 0.001
Septal	1.0 ± 0.12	*P* = 0.007
Diastolic:		ANOVA: F = 51.9, *p* < 0.001
Lateral	0.58 ± 0.13	Reference
Anterior	0.72 ± 0.12	*p* <0.001
Inferior	0.61 ± 0.13	*p* =1.0
Septal	0.83 ± 0.14	*p* <0.001

Values are Bonferroni corrected and therefore a p value of *p* < 0.05 was taken as the level of significance

#### Secondary eigenvector angle

**Fig. 4:**
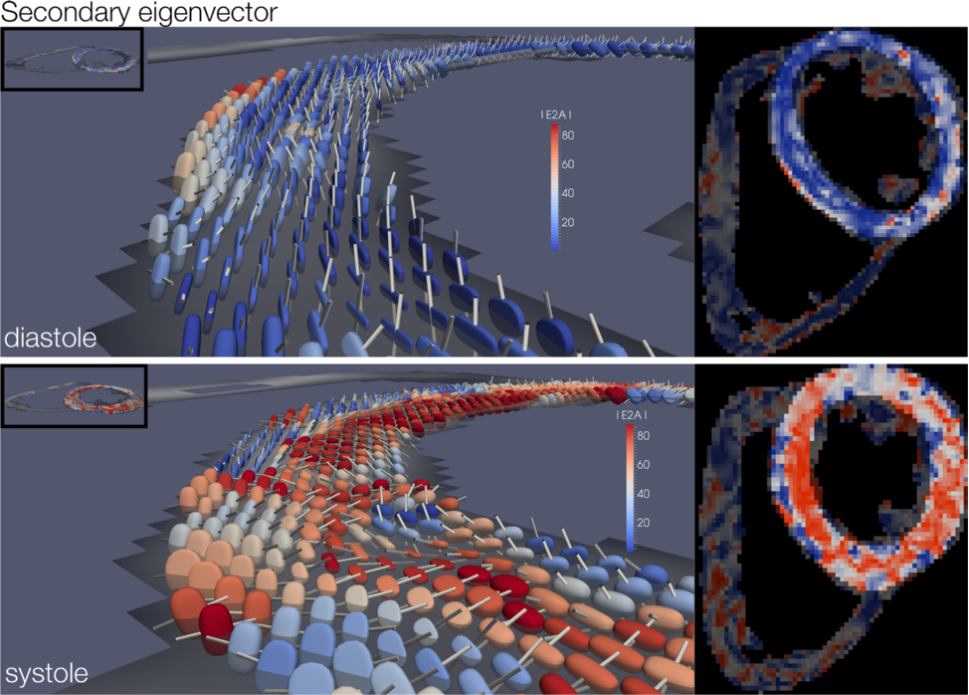
Example parameter maps of E2A in diastole and systole showing re-orientation from a vertical arrangement in diastole (*blue*) to a more horizontal orientation in systole (*orange*-*red*)

E2A angles and E2A mobility in both cardiac phases showed significant between slice variation with greatest angulation in the basal slice (Systole: apical 47 ± 6°, mid 54 ± 6° , basal 58 ± 4° *p* < 0.001; Diastole: apical 23 ± 5° , mid 26 ± 6° , basal 29 ± 6° *p* < 0.001; Mobility: apical 24 ± 9°, mid 27 ± 8°, basal 29 ± 7°, p=0.006). Regional systolic and diastolic, mid slice E2A values are given in Table 5. E2A in systole and diastole is represented in 3D in Fig. 4. Global systolic E2A was 54±6°. Systolic E2A was greater in the lateral wall compared to the anterior wall(*p* = 0.019). Global diastolic E2A was 26 ± 6°. Regional diastolic E2A was less in the lateral wall compared to the inferior (*p* < 0.001) and septal walls (*p* = 0.002). Global E2A mobility was 27 ± 8°. Regionally E2A mobility was greater in the lateral wall compared to the inferior wall (*p* = 0.009).

**Table 5:** Regional E2A (°) in systole and diastole and regional secondary eigenvector mobility, mean ± SD. Data is from the mid ventricular slice only

Secondary Eigenvector Angle (°)	*N* = 43	*p* value
Systolic:		ANOVA: F = 5.07, *p* = 0.02
Lateral	55 ± 8	Reference
Anterior	51 ± 8	*p* = 0.019
Inferior	55 ± 7	*p* = 1.0
Septal	55 ± 10	*p* = 1.0
Diastolic:		ANOVA: F = 16.8, *p* < 0.001
Lateral	24 ± 8	Reference
Anterior	22 ± 8	*p* =1.0
Inferior	30 ± 7	*p* <0.001
Septal	30 ± 10	*p* =0.002
Mobility:		ANOVA: F = 4.64, *p* = 0.004
Lateral	31 ± 11	Reference
Anterior	29 ± 11	*p* = 0.43
Inferior	24 ± 10	*p* = 0.009
Septal	26 ± 14	*p* = 0.11

Values are Bonferroni corrected and a p value of *p* < 0.05 was taken as the level of significance

### Relationship between subject anthropometrics and cDTI Parameters

#### Fractional anisotropy

There was a significant relationship between global systolic FA and RR interval with a decrease in FA of 0.013 for every 100 ms increase in RR (Standardised coefficient −0.43, *p* = 0.004). No subject characteristics were associated with global diastolic FA.

#### Mean diffusivity

Diastolic MD was 0.078 × 10^3^ mm^2^/s less in male patients (Standardised coefficient −0.31, *p* = 0.046). RR interval was inversely associated with diastolic MD (Standardised coefficient −0.30, *p* = 0.048), with a 100 ms increase in RR interval associated with 0.024 × 10^3^ mm^2^/s decrease in MD. LV wall thickness was also inversely related to diastolic MD (−0.37, *p* = 0.015). There were no significant associations between subject anthropometrics and global systolic MD.

#### Helix angle gradient

Global systolic HAg was directly related to RR interval (Standardised coefficient 0.51, *p* < 0.001), with a 100 ms increase in RR interval accounting for an increase in HA between the endo and epicardium of 3°. There was also a direct relationship between systolic HAg and LV end diastolic volume (EDV) (Standardised coefficient 0.51, *p* < 0.001), LV end systolic volume (ESV) (Standardised coefficient 0.33, *p* = 0.033) & LV mass (Standardised coefficient 0.35, *p* = 0.021). Global diastolic HAg was 0.11°/% greater in male patients, *p* < 0.001. Global diastolic HAg was also directly related to BSA (Standardised coefficient 0.44, *p* = 0.003), LVESV (Standardised coefficient 0.33, *p* = 0.031), LV Mass (Standardised coefficient 0.37, *p* = 0.015), and maximum wall thickness in diastole (MWTd) (Standardised coefficient 0.33, *p* = 0.029).

Global systolic and diastolic HAG data were also regressed against subject anthropometrics. Systolic HAG was 0.41°/mm less in male patients, *p* = 0.005. HAG was inversely related to BSA (Standardised coefficient −0.39, *p* = 0.01), LVEDV (Standardised coefficient −0.36, *p* = 0.019), LVMass (Standardised coefficient −0.54, *p* < 0.001), MWTd (Standardised coefficient −0.57, *p* < 0.001). Global diastolic HAG was inversely related to age (Standardised coefficient −0.50, *p* = 0.001), EF (Standardised coefficient −0.37, p = 0.014) and MWTd (Standardised coefficient −0.45, *p* = 0.002).

#### Secondary eigenvector angle

Global systolic E2A was directly related to the RR interval, with an increase in RR of 100 ms associated with an increase in systolic E2A of 1° (*p* = 0.011). Systolic E2A was also related to BSA (Standardised coefficient 0.36, *p* = 0.019), LV Mass (Standardised coefficient 0. 49, *p* = 0.001), LVEDV (Standardised coefficient 0.31, *p* = 0.044) and MWTd (Standardised coefficient 0.48, *p* = 0.001). There were no associations between subject anthropometrics and global diastolic E2A. E2A mobility was inversely related to both age (Standardised coefficient −0.40, *p* = 0.008) and ejection fraction (Standardised coefficient 0.45, *p* = 0.002).

## Discussion

This work is one of  the largest healthy volunteer in vivo cDTI study to date, and the first to study the relationship between subject anthropometrics and quantitative cardiac parameters. This is important because without a clear understanding of normal variation and clinical factors that may affect the cDTI measurements, it is not possible to interpret findings in pathological states with any confidence.

### Global and regional analysis

The global systolic (0.47 ± 0.05) and diastolic (0.56 ± 0.04) FA values for this cohort are largely in keeping with previously published data acquired at 3 T with the *b* = 350 s/mm^2^ STEAM sequence: Nielles-Vallespin (systolic: 0.46 ± 0.04) [42], Tunnicliffe (systolic: 0.41 ± 0.05, diastolic: 0.54 ± 0.04) [28]. We also report subtle but significant regional variation in FA in both cardiac phases, this contrasts with the findings, in healthy subjects, of Tseng et al. [29], and our findings in hypertrophic cardiomyopathy [27] and in healthy volunteers at higher b-values [43]. Regional FA variation (5% in systole, 12% in diastole) appeared clinically insignificant, yet exceeded differences between infarct and remote myocardium previously reported by Wu et al. [30]. We suggest that our observations are most likely due to technical factors such as variation in post processing techniques [28] and regional variations in SNR. One other potential cause of similar FA values on the lateral and septal walls but differing from the values in the anterior and inferior walls is the artefacts caused by off-resonant spins. These artefacts most commonly occur in the phase encoding direction, which is usually most closely aligned with the anterior-posterior direction. However, the orientation of the heart varies on a patient-by-patient basis and similar patterns of heterogeneity would have been present in our previous work if this had been a substantial contributor.

Global phasic results for MD also concur with previous studies adopting this sequence [28, 42]. Regional variation was detected in diastole, with least diffusivity in the anterior wall. Again technical factors are likely to have, in part, contributed to the observed change, including regional SNR heterogeneity and the limited number pixels across the myocardium in diastole. We observed a regional variation of ~18% in diastolic MD, which is greater than the difference between infarct and remote zone reported by Wu et al.(*b* = 300 s/mm^2^) [30, 31], but significantly less than the difference between infarct and remote zone reported by Nyguen et al. (*b* = 400 s/mm^2^) [44]. As MD is the least reproducible of the cDTI parameters [27], caution must therefore be taken when interpreting MD results at lower b values.

There remains a lack of consensus with regard to optimal helix angle presentation. In this work, we provide descriptive data of the angle by region and transmural layer, in addition to statistical comparison of regional helix angle gradient (HAg), acquired from best-fit, radial transmural projections. The advantage of the latter reduced sensitivity to ROI variation, but this is at the expense of data smoothing. The systolic obliquity of myocyte helices and radial reorientation of sheetlet angles are becoming increasing recognised as important components of LV shortening and myocardial thickening respectively [6, 8–10, 22, 45–47]. This work provides further insights into regional variation of these structures. The observed regional variation in HAg appears, partially attributable to the innate heterogeneity of wall thickness, with the steepest gradients observed in the septum. Despite data acquisition from 3 minimally spaced slices, E2A angles, in both cardiac phases, echoed results from Stoeck et al. [9], increasing significantly from the most apical slice to the most basal slice. Minor regional variation of uncertain significance was observed in diastolic E2A and E2A mobility with greatest mobility in the lateral wall.

### Relationship between subject anthropometrics and cDTI Parameters

There have been no previous reports relating quantitative measures of cardiac cDTI to patient anthropometrics. This is important to understand, because the complex factors that affect DTI measurements may be affected by simple clinical variables. We observed that, despite applying heart rate correction to the b-value, there was residual heart rate dependent variability within global systolic FA, HAg and E2A measurements. However this effect was clinically small, with variation in parameters over a heart rate range of 60–100 bpm of 14° in epicardial to endocardial helix angle, 0.05 in FA, and 4° in systolic E2A. The reason behind this effect is unclear, but may be related to heart rate dependent changes in strain, or shorter mixing times at faster heart rates.

As might be expected global systolic and diastolic HAg is directly related to BSA, LVEDV, LV mass and male gender. Interestingly, many of these correlations persist when helix angle gradient is normalised to the local wall thickness (HAG). This indicates that helix angle has neither a constant range independent of myocardial thickness, nor does it have a constant absolute gradient in degrees/mm.

Diastolic E2A conformation was independent of subject characteristics, but in systole increasing BSA, LV mass and LV wall thickness was associated with increasing radial orientation (an increase in LV mass of 50 g or BSA of 0.5 m^2^ accounting for an increase in E2A of 4° and a 1 mm increment in wall thickness accounting for an increase of 2°). With regards to sheetlet mobility: within this healthy population with normal range ejection fractions, an increase in EF of 10% was associated with a decrease in E2A mobility of 5°. Initially this seems somewhat counter intuitive as sheetlet mobility is perceived to be an integral component of LV function. However, increasing age was also associated with decreasing E2A mobility and age related changes in LV function and volumes are well documented, with increasing EF and decreasing cardiac volumes with increasing age [40, 41]. The inverse relationship between E2A mobility and ejection fraction is therefore likely a reflection of the artificial age related increase in this measurement. Nonetheless, clinically these observations are relatively minor compared to disease related changes; with a difference in E2A mobility on average of 15° between patients with hypertrophic cardiomyopathy and healthy volunteers [8].

### Limitations

There are a number of limitations of this work, including the inherently low SNR and spatial resolution of the STEAM diffusion sequence; the possibly of misregistration of slice averages, which could be age or gender dependent; and the potential for strain artefacts. We did not attempt strain correction of our results as we believe that contemporary strain measures are flawed by the assumption that the myocardium behaves like an isotropic jelly. The true impact of strain on quantitative cDTI parameters is a matter of significant debate in this field [8, 9, 45, 48, 49]. However, it remains that strain may have, in part, contributed to the observed phasic heterogeneity, as well as gender and age related associations [50, 51].

## Conclusion

Quantitative cDTI parameters display anthropometric variation, most notably HAg which increases with cardiac size. The contribution of technical factors to regional parameter heterogeneity must be taken into consideration when making clinical interpretations at lower b values.

## Abbreviations

bSSFP: balanced steady state free precession
BSA body surface area cDTI: cardiac diffusion tensor imaging
CMR: cardiovascular magnetic resonance
DW STEAM: diffusion weighted stimulated echo sequence
E2A: secondary eigenvector angle
EF ejection fraction EDV: end diastolic volume
ESV: end systolic volume
FA: fractional anisotropy
GRE: gradient echo
HA: helical angle
HAg (degrees/%depth) HAG: helix angle gradient(degrees/mm)
LV: left ventricle
MD: mean diffusivity
MWTd: maximum wall thickness in diastole
SNR: signal to noise ration
SPAMM: spatial modulation of magnetization
STEAM: stimulated echo sequence
